# Supplementary figures and images for: Detecting Parkinson’s disease from shoe-mounted accelerometer sensors using convolutional neural networks optimized with modified metaheuristics (part 1 of 2)
Source: PeerJ Comput Sci. 2024 May 13;10:e2031. doi: 10.7717/peerj-cs.2031 (PMC11157549; doi:10.7717/peerj-cs.2031)

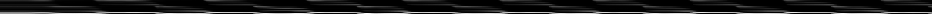

Supplement: Supplemental Information 2 [file peerj-cs-10-2031-s002.zip › Out/GaCo01_01_part_1.png]

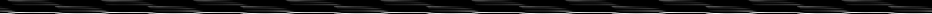

Supplement: Supplemental Information 2 [file peerj-cs-10-2031-s002.zip › Out/GaCo01_01_part_10.png]

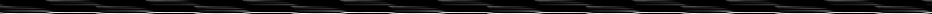

Supplement: Supplemental Information 2 [file peerj-cs-10-2031-s002.zip › Out/GaCo01_01_part_2.png]

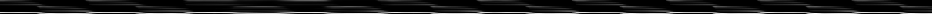

Supplement: Supplemental Information 2 [file peerj-cs-10-2031-s002.zip › Out/GaCo01_01_part_3.png]

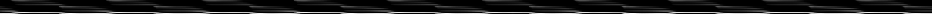

Supplement: Supplemental Information 2 [file peerj-cs-10-2031-s002.zip › Out/GaCo01_01_part_4.png]

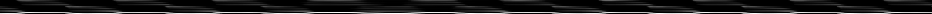

Supplement: Supplemental Information 2 [file peerj-cs-10-2031-s002.zip › Out/GaCo01_01_part_5.png]

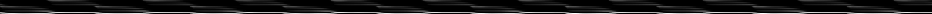

Supplement: Supplemental Information 2 [file peerj-cs-10-2031-s002.zip › Out/GaCo01_01_part_6.png]

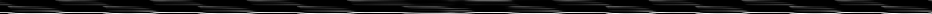

Supplement: Supplemental Information 2 [file peerj-cs-10-2031-s002.zip › Out/GaCo01_01_part_7.png]

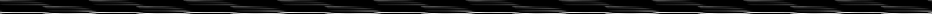

Supplement: Supplemental Information 2 [file peerj-cs-10-2031-s002.zip › Out/GaCo01_01_part_8.png]

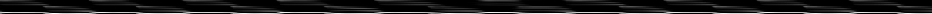

Supplement: Supplemental Information 2 [file peerj-cs-10-2031-s002.zip › Out/GaCo01_01_part_9.png]

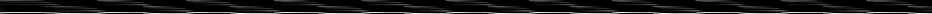

Supplement: Supplemental Information 2 [file peerj-cs-10-2031-s002.zip › Out/GaCo02_01_part_1.png]

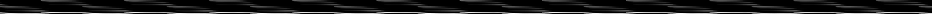

Supplement: Supplemental Information 2 [file peerj-cs-10-2031-s002.zip › Out/GaCo02_01_part_10.png]

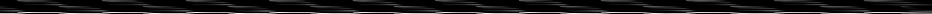

Supplement: Supplemental Information 2 [file peerj-cs-10-2031-s002.zip › Out/GaCo02_01_part_2.png]

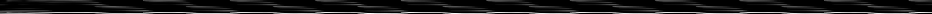

Supplement: Supplemental Information 2 [file peerj-cs-10-2031-s002.zip › Out/GaCo02_01_part_3.png]

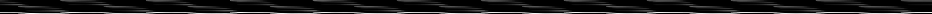

Supplement: Supplemental Information 2 [file peerj-cs-10-2031-s002.zip › Out/GaCo02_01_part_4.png]

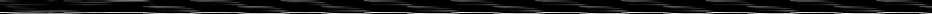

Supplement: Supplemental Information 2 [file peerj-cs-10-2031-s002.zip › Out/GaCo02_01_part_5.png]

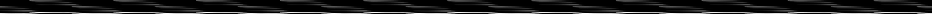

Supplement: Supplemental Information 2 [file peerj-cs-10-2031-s002.zip › Out/GaCo02_01_part_6.png]

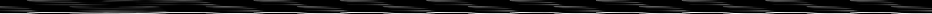

Supplement: Supplemental Information 2 [file peerj-cs-10-2031-s002.zip › Out/GaCo02_01_part_7.png]

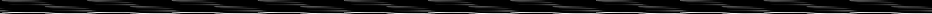

Supplement: Supplemental Information 2 [file peerj-cs-10-2031-s002.zip › Out/GaCo02_01_part_8.png]

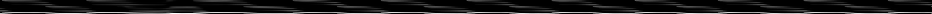

Supplement: Supplemental Information 2 [file peerj-cs-10-2031-s002.zip › Out/GaCo02_01_part_9.png]

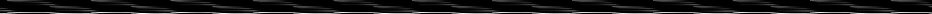

Supplement: Supplemental Information 2 [file peerj-cs-10-2031-s002.zip › Out/GaCo02_02_part_1.png]

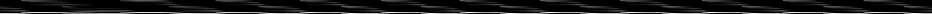

Supplement: Supplemental Information 2 [file peerj-cs-10-2031-s002.zip › Out/GaCo02_02_part_10.png]

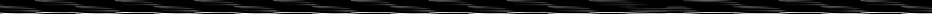

Supplement: Supplemental Information 2 [file peerj-cs-10-2031-s002.zip › Out/GaCo02_02_part_2.png]

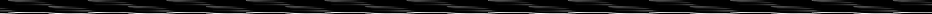

Supplement: Supplemental Information 2 [file peerj-cs-10-2031-s002.zip › Out/GaCo02_02_part_3.png]

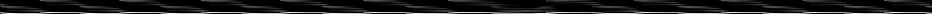

Supplement: Supplemental Information 2 [file peerj-cs-10-2031-s002.zip › Out/GaCo02_02_part_4.png]

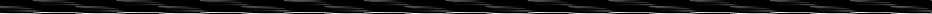

Supplement: Supplemental Information 2 [file peerj-cs-10-2031-s002.zip › Out/GaCo02_02_part_5.png]

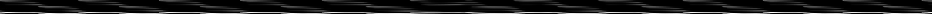

Supplement: Supplemental Information 2 [file peerj-cs-10-2031-s002.zip › Out/GaCo02_02_part_6.png]

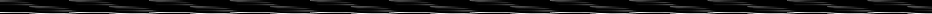

Supplement: Supplemental Information 2 [file peerj-cs-10-2031-s002.zip › Out/GaCo02_02_part_7.png]

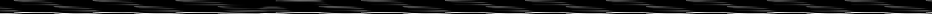

Supplement: Supplemental Information 2 [file peerj-cs-10-2031-s002.zip › Out/GaCo02_02_part_8.png]

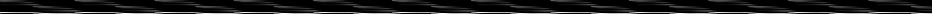

Supplement: Supplemental Information 2 [file peerj-cs-10-2031-s002.zip › Out/GaCo02_02_part_9.png]

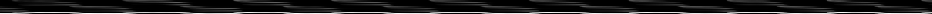

Supplement: Supplemental Information 2 [file peerj-cs-10-2031-s002.zip › Out/GaCo03_01_part_1.png]

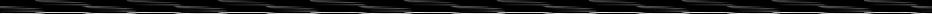

Supplement: Supplemental Information 2 [file peerj-cs-10-2031-s002.zip › Out/GaCo03_01_part_10.png]

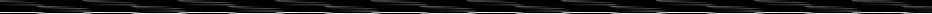

Supplement: Supplemental Information 2 [file peerj-cs-10-2031-s002.zip › Out/GaCo03_01_part_2.png]

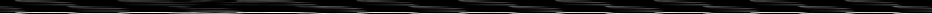

Supplement: Supplemental Information 2 [file peerj-cs-10-2031-s002.zip › Out/GaCo03_01_part_3.png]

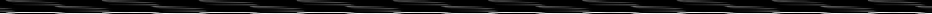

Supplement: Supplemental Information 2 [file peerj-cs-10-2031-s002.zip › Out/GaCo03_01_part_4.png]

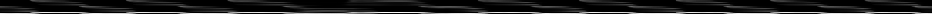

Supplement: Supplemental Information 2 [file peerj-cs-10-2031-s002.zip › Out/GaCo03_01_part_5.png]

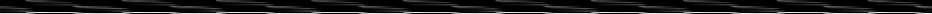

Supplement: Supplemental Information 2 [file peerj-cs-10-2031-s002.zip › Out/GaCo03_01_part_6.png]

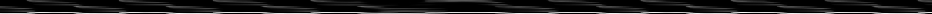

Supplement: Supplemental Information 2 [file peerj-cs-10-2031-s002.zip › Out/GaCo03_01_part_7.png]

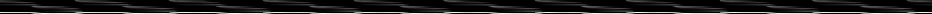

Supplement: Supplemental Information 2 [file peerj-cs-10-2031-s002.zip › Out/GaCo03_01_part_8.png]

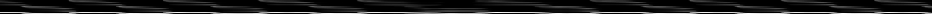

Supplement: Supplemental Information 2 [file peerj-cs-10-2031-s002.zip › Out/GaCo03_01_part_9.png]

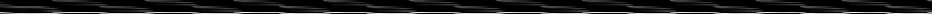

Supplement: Supplemental Information 2 [file peerj-cs-10-2031-s002.zip › Out/GaCo03_02_part_1.png]

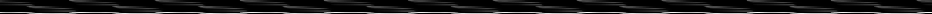

Supplement: Supplemental Information 2 [file peerj-cs-10-2031-s002.zip › Out/GaCo03_02_part_10.png]

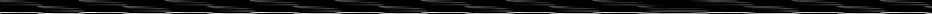

Supplement: Supplemental Information 2 [file peerj-cs-10-2031-s002.zip › Out/GaCo03_02_part_2.png]

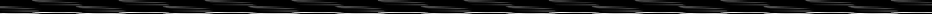

Supplement: Supplemental Information 2 [file peerj-cs-10-2031-s002.zip › Out/GaCo03_02_part_3.png]

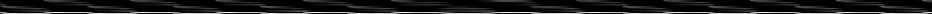

Supplement: Supplemental Information 2 [file peerj-cs-10-2031-s002.zip › Out/GaCo03_02_part_4.png]

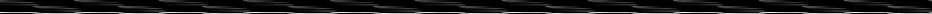

Supplement: Supplemental Information 2 [file peerj-cs-10-2031-s002.zip › Out/GaCo03_02_part_5.png]

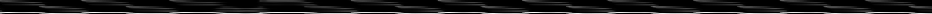

Supplement: Supplemental Information 2 [file peerj-cs-10-2031-s002.zip › Out/GaCo03_02_part_6.png]

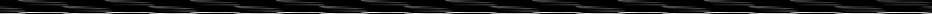

Supplement: Supplemental Information 2 [file peerj-cs-10-2031-s002.zip › Out/GaCo03_02_part_7.png]

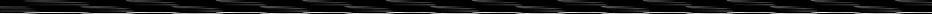

Supplement: Supplemental Information 2 [file peerj-cs-10-2031-s002.zip › Out/GaCo03_02_part_8.png]

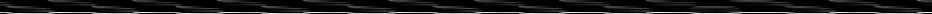

Supplement: Supplemental Information 2 [file peerj-cs-10-2031-s002.zip › Out/GaCo03_02_part_9.png]

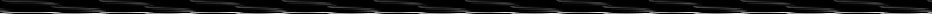

Supplement: Supplemental Information 2 [file peerj-cs-10-2031-s002.zip › Out/GaCo04_01_part_1.png]

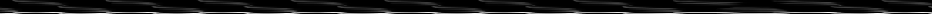

Supplement: Supplemental Information 2 [file peerj-cs-10-2031-s002.zip › Out/GaCo04_01_part_10.png]

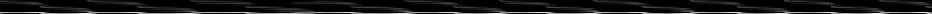

Supplement: Supplemental Information 2 [file peerj-cs-10-2031-s002.zip › Out/GaCo04_01_part_2.png]

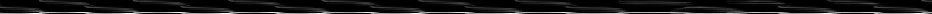

Supplement: Supplemental Information 2 [file peerj-cs-10-2031-s002.zip › Out/GaCo04_01_part_3.png]

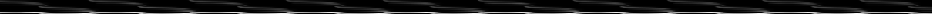

Supplement: Supplemental Information 2 [file peerj-cs-10-2031-s002.zip › Out/GaCo04_01_part_4.png]

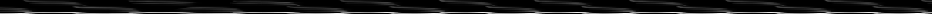

Supplement: Supplemental Information 2 [file peerj-cs-10-2031-s002.zip › Out/GaCo04_01_part_5.png]

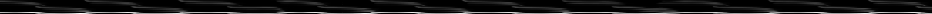

Supplement: Supplemental Information 2 [file peerj-cs-10-2031-s002.zip › Out/GaCo04_01_part_6.png]

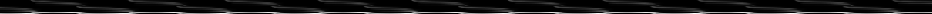

Supplement: Supplemental Information 2 [file peerj-cs-10-2031-s002.zip › Out/GaCo04_01_part_7.png]

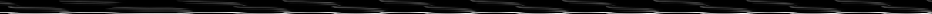

Supplement: Supplemental Information 2 [file peerj-cs-10-2031-s002.zip › Out/GaCo04_01_part_8.png]

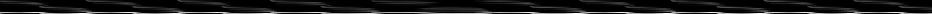

Supplement: Supplemental Information 2 [file peerj-cs-10-2031-s002.zip › Out/GaCo04_01_part_9.png]

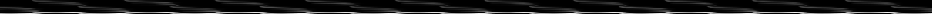

Supplement: Supplemental Information 2 [file peerj-cs-10-2031-s002.zip › Out/GaCo04_02_part_1.png]

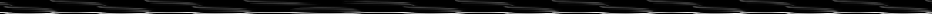

Supplement: Supplemental Information 2 [file peerj-cs-10-2031-s002.zip › Out/GaCo04_02_part_10.png]

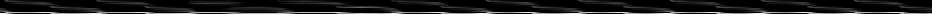

Supplement: Supplemental Information 2 [file peerj-cs-10-2031-s002.zip › Out/GaCo04_02_part_2.png]

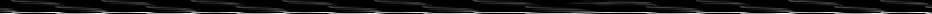

Supplement: Supplemental Information 2 [file peerj-cs-10-2031-s002.zip › Out/GaCo04_02_part_3.png]

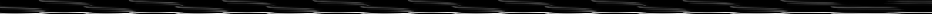

Supplement: Supplemental Information 2 [file peerj-cs-10-2031-s002.zip › Out/GaCo04_02_part_4.png]

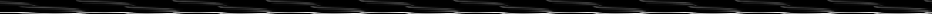

Supplement: Supplemental Information 2 [file peerj-cs-10-2031-s002.zip › Out/GaCo04_02_part_5.png]

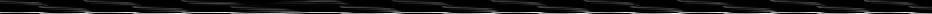

Supplement: Supplemental Information 2 [file peerj-cs-10-2031-s002.zip › Out/GaCo04_02_part_6.png]

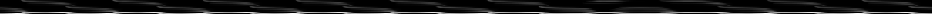

Supplement: Supplemental Information 2 [file peerj-cs-10-2031-s002.zip › Out/GaCo04_02_part_7.png]

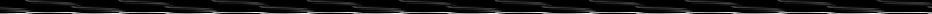

Supplement: Supplemental Information 2 [file peerj-cs-10-2031-s002.zip › Out/GaCo04_02_part_8.png]

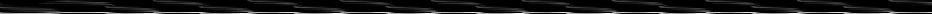

Supplement: Supplemental Information 2 [file peerj-cs-10-2031-s002.zip › Out/GaCo04_02_part_9.png]

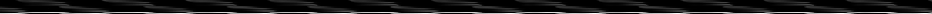

Supplement: Supplemental Information 2 [file peerj-cs-10-2031-s002.zip › Out/GaCo05_01_part_1.png]

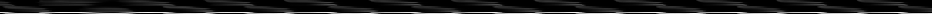

Supplement: Supplemental Information 2 [file peerj-cs-10-2031-s002.zip › Out/GaCo05_01_part_10.png]

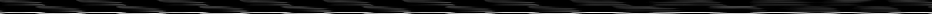

Supplement: Supplemental Information 2 [file peerj-cs-10-2031-s002.zip › Out/GaCo05_01_part_2.png]

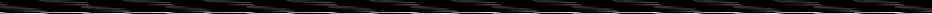

Supplement: Supplemental Information 2 [file peerj-cs-10-2031-s002.zip › Out/GaCo05_01_part_3.png]

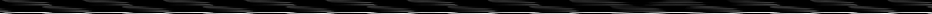

Supplement: Supplemental Information 2 [file peerj-cs-10-2031-s002.zip › Out/GaCo05_01_part_4.png]

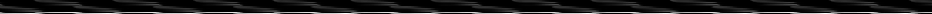

Supplement: Supplemental Information 2 [file peerj-cs-10-2031-s002.zip › Out/GaCo05_01_part_5.png]

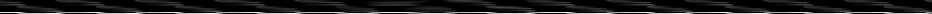

Supplement: Supplemental Information 2 [file peerj-cs-10-2031-s002.zip › Out/GaCo05_01_part_6.png]

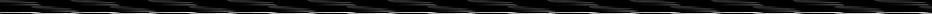

Supplement: Supplemental Information 2 [file peerj-cs-10-2031-s002.zip › Out/GaCo05_01_part_7.png]

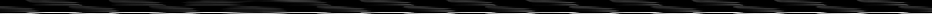

Supplement: Supplemental Information 2 [file peerj-cs-10-2031-s002.zip › Out/GaCo05_01_part_8.png]

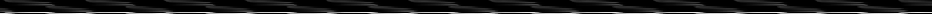

Supplement: Supplemental Information 2 [file peerj-cs-10-2031-s002.zip › Out/GaCo05_01_part_9.png]

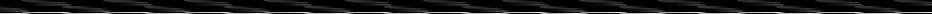

Supplement: Supplemental Information 2 [file peerj-cs-10-2031-s002.zip › Out/GaCo05_02_part_1.png]

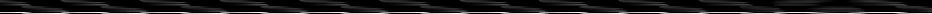

Supplement: Supplemental Information 2 [file peerj-cs-10-2031-s002.zip › Out/GaCo05_02_part_10.png]

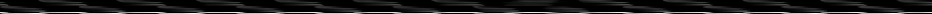

Supplement: Supplemental Information 2 [file peerj-cs-10-2031-s002.zip › Out/GaCo05_02_part_2.png]

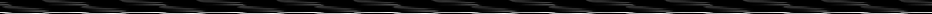

Supplement: Supplemental Information 2 [file peerj-cs-10-2031-s002.zip › Out/GaCo05_02_part_3.png]

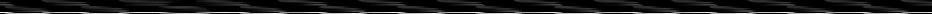

Supplement: Supplemental Information 2 [file peerj-cs-10-2031-s002.zip › Out/GaCo05_02_part_4.png]

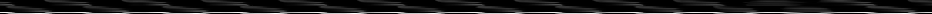

Supplement: Supplemental Information 2 [file peerj-cs-10-2031-s002.zip › Out/GaCo05_02_part_5.png]

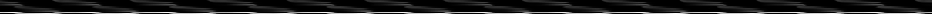

Supplement: Supplemental Information 2 [file peerj-cs-10-2031-s002.zip › Out/GaCo05_02_part_6.png]

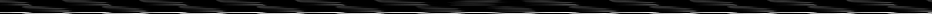

Supplement: Supplemental Information 2 [file peerj-cs-10-2031-s002.zip › Out/GaCo05_02_part_7.png]

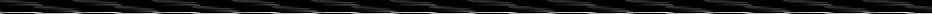

Supplement: Supplemental Information 2 [file peerj-cs-10-2031-s002.zip › Out/GaCo05_02_part_8.png]

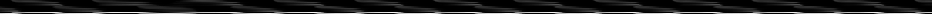

Supplement: Supplemental Information 2 [file peerj-cs-10-2031-s002.zip › Out/GaCo05_02_part_9.png]

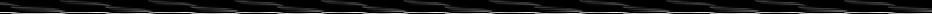

Supplement: Supplemental Information 2 [file peerj-cs-10-2031-s002.zip › Out/GaCo06_01_part_1.png]

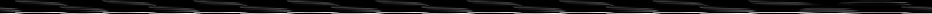

Supplement: Supplemental Information 2 [file peerj-cs-10-2031-s002.zip › Out/GaCo06_01_part_10.png]

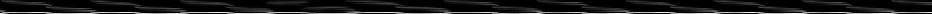

Supplement: Supplemental Information 2 [file peerj-cs-10-2031-s002.zip › Out/GaCo06_01_part_2.png]

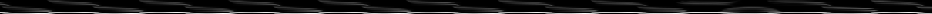

Supplement: Supplemental Information 2 [file peerj-cs-10-2031-s002.zip › Out/GaCo06_01_part_3.png]

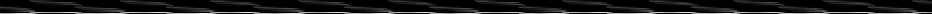

Supplement: Supplemental Information 2 [file peerj-cs-10-2031-s002.zip › Out/GaCo06_01_part_4.png]

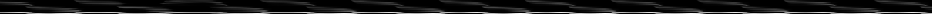

Supplement: Supplemental Information 2 [file peerj-cs-10-2031-s002.zip › Out/GaCo06_01_part_5.png]

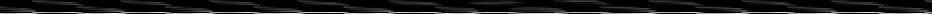

Supplement: Supplemental Information 2 [file peerj-cs-10-2031-s002.zip › Out/GaCo06_01_part_6.png]

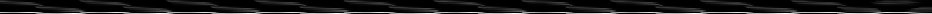

Supplement: Supplemental Information 2 [file peerj-cs-10-2031-s002.zip › Out/GaCo06_01_part_7.png]

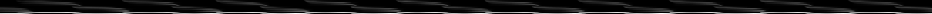

Supplement: Supplemental Information 2 [file peerj-cs-10-2031-s002.zip › Out/GaCo06_01_part_8.png]

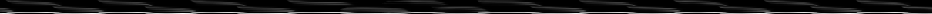

Supplement: Supplemental Information 2 [file peerj-cs-10-2031-s002.zip › Out/GaCo06_01_part_9.png]
